# Diversity and Taxonomy of Endophytic Xylariaceous Fungi from Medicinal Plants of *Dendrobium* (Orchidaceae)

**DOI:** 10.1371/journal.pone.0058268

**Published:** 2013-03-05

**Authors:** Juan Chen, Li-Chun Zhang, Yong-Mei Xing, Yun-Qiang Wang, Xiao-Ke Xing, Da-Wei Zhang, Han-Qiao Liang, Shun-Xing Guo

**Affiliations:** 1 Institute of Medicinal Plant Development, Chinese Academy of Medical Sciences & Peking Union Medical College, Beijing, People’s Republic of China; 2 Botanical Garden of Xishuangbanna South Medicine, Jinghong, Yunnan, People’s Republic of China; Université Paris-Sud, France

## Abstract

*Dendrobium* spp. are traditional Chinese medicinal plants, and the main effective ingredients (polysaccharides and alkaloids) have pharmacologic effects on gastritis infection, cancer, and anti-aging. Previously, we confirmed endophytic xylariaceous fungi as the dominant fungi in several *Dendrobium* species of tropical regions from China. In the present study, the diversity, taxonomy, and distribution of culturable endophytic xylariaceous fungi associated with seven medicinal species of *Dendrobium* (Orchidaceae) were investigated. Among the 961 endophytes newly isolated, 217 xylariaceous fungi (morphotaxa) were identified using morphological and molecular methods. The phylogenetic tree constructed using nuclear ribosomal internal transcribed spacer (ITS), large subunit of ribosomal DNA (LSU), and beta-tubulin sequences divided these anamorphic xylariaceous isolates into at least 18 operational taxonomic units (OTUs). The diversity of the endophytic xylariaceous fungi in these seven *Dendrobium* species was estimated using Shannon and evenness indices, with the results indicating that the dominant Xylariaceae taxa in each *Dendrobium* species were greatly different, though common xylariaceous fungi were found in several *Dendrobium* species. These findings implied that different host plants in the same habitats exhibit a preference and selectivity for their fungal partners. Using culture-dependent approaches, these xylariaceous isolates may be important sources for the future screening of new natural products and drug discovery.

## Introduction

Since the discovery of Taxol, an anticancer drug from endophytic fungi, such as *Taxomyces andreanae, Pestalotia* spp. and *Pestalotiopsis* spp. [1], [2], endophytes as novel sources of phytochemicals and bioactive substances with promising medicinal and agricultural application have attracted much attention from mycologists and chemists worldwide [3]–[5]. Endophytic fungi can directly enhance plant growth, increase the host plants’ adaptability to such diverse conditions as dry, cold, and high-salt environments, and help to resist pathogen damage [6], as well as being able to alter the community structure [7] and antioxidant activity of the host plants [8]. Recently, based on their good antimicrobial activity, more attention has been given to the potential of exploiting endophytic fungi for novel antibiotics [9]–[11]. Endophytic fungi are ubiquitous and diverse in plants, and it is estimated that there are approximately 1 million species worldwide [12]; however, only a fraction has been described and explored to date. Although the complex relationship between endophytic fungi and saprophytic and pathogenic fungi has been studied [12]–[14], their authentic function in ecological systems remains elusive.

Xylariaceae is one of the largest and most widely distributed families of Xylariales (Ascomycota), with approximately 85 genera and at least 1340 species [15]. The members of this family are usually considered to be saprophytic, but some taxa have been described as endophytic or pathogenic [16], [17], with a few species being associated with termites [18]. In the past thirty years, a number of studies have reported on the taxonomy, phylogenetic reconstruction, ecology, and relationship between xylariaceous fungal teleomorph and anamorph stages, including work on endophytic xylariaceous fungi [19]–[28]. Moreover, a series of new compounds have been isolated from Xylariaceae species and demonstrated good antimicrobial or anticancer bioactivity, particularly endophytic *Xylaria* [29]–[32]. Endophytic xylariaceous fungi were also recently investigated from many herbs and woody plants, such as liverworts [33], *Lepanthes* and *Dendrobium* (Orchidaceae) [34], [35], *Piper* (Piperaceae) [36], *Pinus* and *Picea* (Pinaceae) [37] and mangroves [32]. In a previous study, we assessed the diversity of endophytic fungi from *Dendrobium* (Orchidaceae) and found that xylariaceous fungi were among the dominant taxa in the roots of tropical epiphytic Orchidaceae plants [38]. However, their diversity, taxonomy and the specificity of their relationship with host plants remains unaddressed. In the present study, the culturable endophytic xylariaceous fungi isolated from *Dendrobium* were examined for their morphological characters and DNA sequences to reveal their diversity and distribution in their host plants. Using a culture-dependent approach, these xylariaceous isolates will be important sources for the future screening of new natural products and drug discovery.

## Results

### Fungal Isolation and Identification

A total of 961 culturable endophytic fungi were isolated from seven species of *Dendrobium*. The isolation rate of endophytic fungi in each plant species is listed in Table 1. Most of the isolates were asexual mycelial fungi and rarely produced diagnostically morphological characters, such as conidia or ascospores and basidiospores, thus DNA sequences were selected as the main tool to identify these fungi. Based on morphological and molecular results, 870 strains were identified as belonging to at least 9 orders, 25 genera, and approximately 60 species. The remaining 91 strains can not be identified to the genus level by DNA sequences because no ITS sequences matching with high similarity in the GenBank database could be found. In the original 961 cultures, the xylariaceous taxa had the highest isolation frequency, 22.58% (217/961), followed by *Fusarium* at 10.71% (103/961), *Colletotrichum* at 6.55% (63/961), and *Phomopsis* at 4.37% (42/961). Thus, xylariaceous fungi as the dominant group were further analyzed.

**Table 1:** The isolation rate of endophytic fungi from each *Dendrobium* species in the study.

Plant taxa	Abbr.	Plant individual	Root samples	Total root segments	Number of isolated fungi	Isolation rate
*D. nobile*	DNO	2	7	269	143	53.1%
*D. fimbriatum*	DFI	3	8	352	166	47.1%
*D. chrysotoxum*	DCO	3	7	274	227	82.8%
*D. faiconeri*	DFA	1	5	170	64	37.6%
*D. chrysanthum*	DCH	3	6	280	164	58.5%
*D. aphyllum*	DAP	2	5	213	59	27.6%
*D. crystallinum*	DCR	1	4	222	138	62.1%
Total	7	15	42	1780	961	

**Figure 1:**
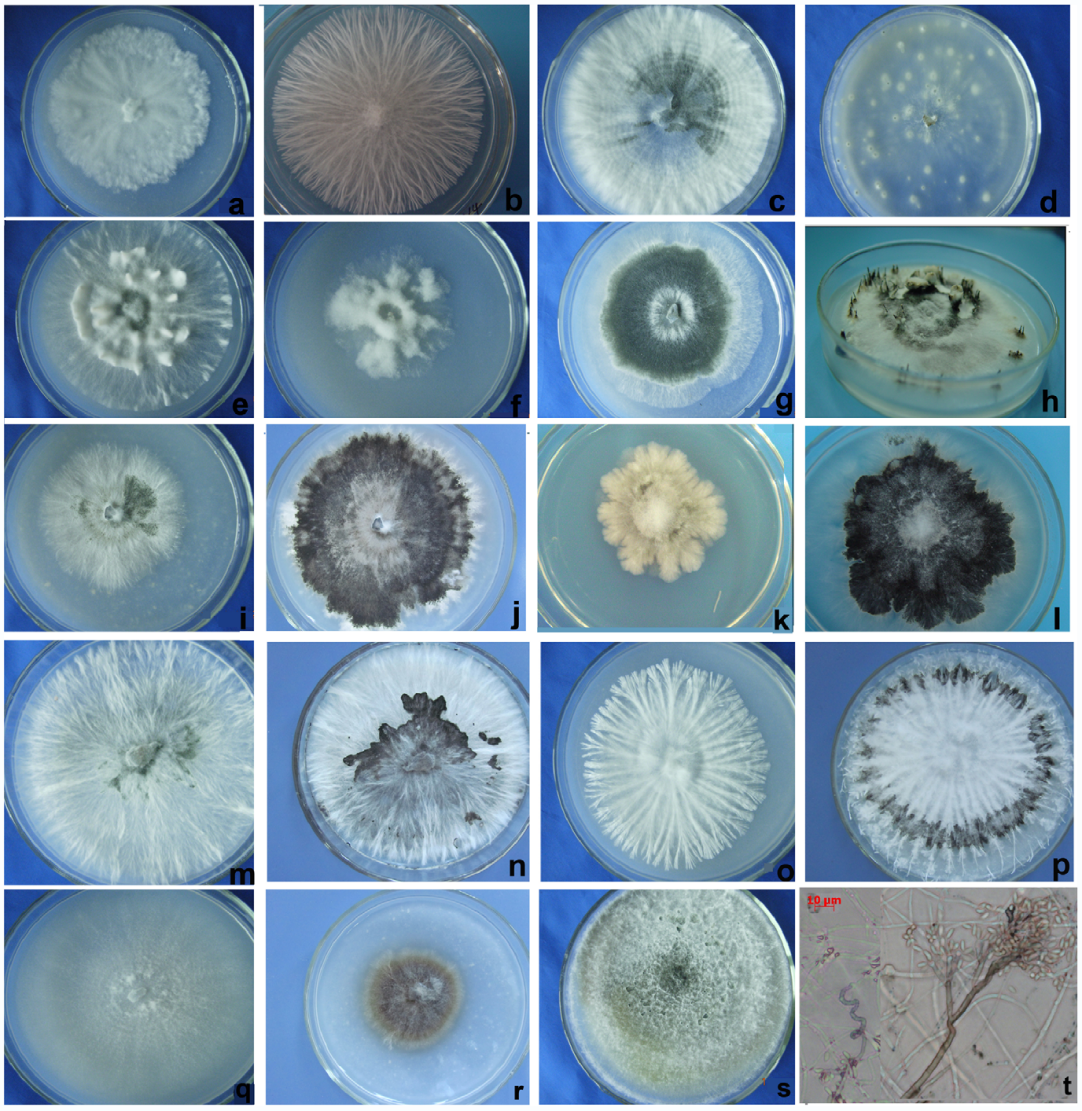
Colony morphology of xylariaceous endophytes on oatmeal agar isolated from *Dendrobium* in this study. **a. 5063** after two weeks; **b. 5054** after two weeks; **c. 5129** after two weeks; **d.** **5268** after two weeks; **e.** **5071** after two weeks; **f. 5311** after two weeks; **g**. **5147** after two weeks; **h. 5147** after four weeks on PDA; **i–j. 5165** after two weeks and five weeks; **k–l.** **5128** after two weeks and five weeks; **m–n**. **5306** after two weeks and five weeks; **o–p. 5089** after two weeks and five weeks; **q. 5327** after two weeks; **r. 5120** after five weeks; **s. 5341** after two weeks; **t.** Coiled hypha, conidiophore and conidia of **5341** after five weeks.

**Figure 2:**
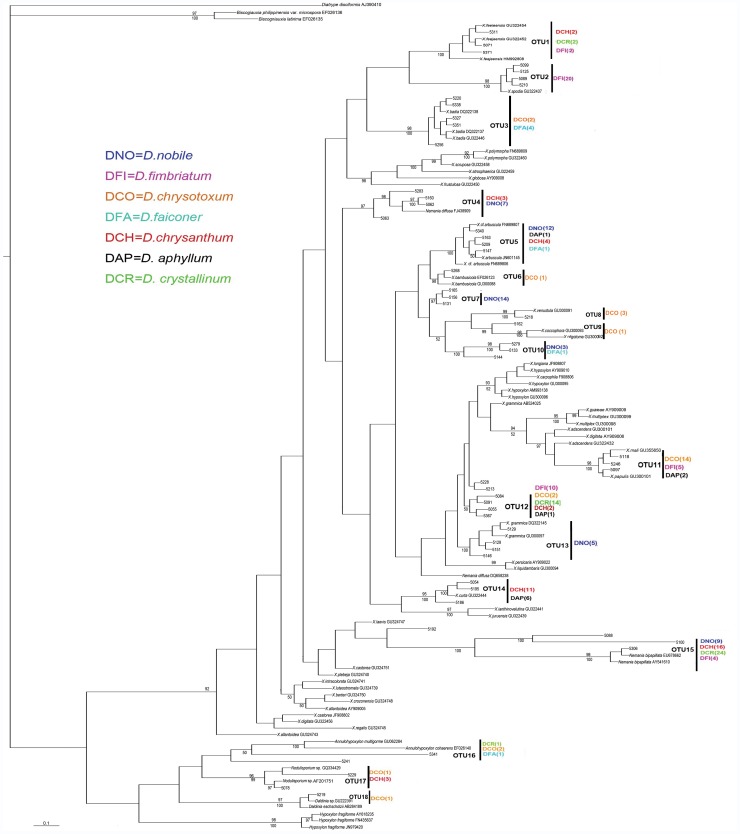
Bayesian 90% majority-rule tree for endophytic Xylariaceae isolated from *Dendrobium* from 5.8S-ITS2 sequences. Values above branches represent posterior probabilities (≥90%) and values below branches are bootstrap values (≥50%) from 1000 replicates. Sequences generated by this study are those numbers with “5” initials.

The morphological examinations of the xylariaceous fungi were performed according to the description of Callan & Rogers [19]–[21]. Some representative strains were incubated on 2.5% oatmeal agar (OA) at 20°C and 12 h of fluorescent light per day for stromatal and conidial production. The newly isolated 217 xylariaceous strains were divided into at least 13 morphotypes based on their cultural characters (e.g., colony color and surface morphology, stromatal production and conidial and conidiophore morphology) (Table 2, Figure 1), and at least 18 possible operational taxonomic units (OTUs) were recognized by phylogenetic analyses (Table 3, Figure 2). The estimation of the Shannon-Weiner diversity index based on the number of OTUs showed that *D. nobile* presented the highest *Xylaria* species diversity among the seven plant species (1.68), followed by *D. chrysanthum* (1.47) and *D. chrysotoxum* (1.46) (Table 3). Furthermore, *D. nobile* showed a higher evenness index (0.94). The similarity index of the Xylariaceae taxa between *D. fimbriatum* and *D. crystallinum* (67%) was higher than that between *D. chrysanthum* and *D. crystallinum* (60%) (Table 4).

**Table 2:** Morphological characters of endophytic xylariaceous fungi.

Morphortype	Representive strains	Culture characters in OA medium						Figure
		Colony diameter (mm, after two weeks)	Colony colors	Colony surface morphology	Colony margins	Stromatal production and morphology	Anamorph morphology (Conidia and Conidiophores)	
Type 1	5063, 5100, 5192	5.5	white	aerial mycelium appressed, abundant	irregular	absent	absent	Figure 1a
Type 2	5054,5186	8.5	white, then black in mature	aerial mycelium felty, abundant	conspicuous radial stripe	absent	absent	Figure 1b
Type 3	5055,5084,5129	9.0	white, then black in center in mature	aerial mycelium scant to abundant,with dense concentrically zonate	entire	absent	absent	Figure 1c
Type 4	5268	9.0	white	aerial mycelium scant, with starmicro-colony	entire	black stromatasubmergingin medium	absent	Figure 1d
Type 5	5071, 5311	9.0	white	aerial mycelium lanose, abundant	conspicuous radial stripe	white, cylindriformyoung stromataproduction	absent	Figure 1e,f
Type 6	5147	9.0	white andblack-greenin center	aerial mycelium appressed abundant	entire	black, cylindriform stromata production	absent	Figure 1g,h
Type 7	5165	5.5	white, then blackin mature	aerial mycelium lanose, abundant	entire	absent	absent	Figure 1i,j
Type 8	5128	5.0	pale yellow, thenblack in mature	aerial mycelium lanose, abundant	plumose	absent	absent	Figure 1k,l
Type 9	5306	9.0	white, then blackin mature	aerial mycelium appressed, abundant	plumose	absent	absent	Figure 1m,n
Type 10	5083,5089, 5099,5125	6.0	white, then black in mature	aerial mycelium lanose, abundant	conspicuous stripe with plumose branch on end	absent	absent	Figure 1o,p
Type 11	5327	9.0	pale white	aerial mycelium submerging, scant	entire	absent	absent	Figure 1q
Type 12	5120	4.0	brown	aerial mycelium lanose, abundant	entire	absent	absent	Figure 1r
Type 13	5341	9.0	pale yellow, then brown in mature	aerial mycelium lanose, abundant	entire	absent	conidia produced from aerialmycelium.	Figure 1s,t

**Table 3:** Distribution of xylariaceous endophytes in the seven *Dendrobium* species in the study.

Fungal isolate	Closet of Genbank match (ITS sequence)			OTUs	*D. nobile*	*D. fimbriatum*	*D. chrysotoxum*	*D. chrysanthum*	*D. falconeri*	*D. aphyllum*	*D.crystallinum*	Total
Representative strains	Referencespecies(RS)	AccesionNo.of RS	Identity(%)									
5088,5306	*Nemania bipapillata*	GU292818	99	OTU15	9	4		16			24	43
5054,5186	*Xylaria curta*	GU322444	97	OTU14				11		6		17
5146,5151,5128	*Xylaria grammica*	GU300097	97	OTU13	5							5
5084,5091,5055,5228	*Xylaria grammica*	GU300097	99	OTU12		10	2	2		1	14	29
5311,5071,5371	*Xylaria feejeensis*	GU322454	99	OTU1		2		2			2	6
5210,5099,5125	*Xylaria apoda*	GU322437	99	OTU2		20						20
5097, 5118	*Xylaria papulis*	GU300100	99	OTU11		5	14			2		21
5147,5163	*Xylaria arbuscula*	GU300090	98	OTU5	12			4	1	1		18
5165,5156,5131	*Xylaria venosula*	GU797434	96	OTU7	14							14
5144,5133,5279	*Xylaria amphithele*	GU300083	92	OTU10	3				1			4
5192	*Nemania primolutea*	EF026121	97	OTU15						3		3
5218,5162	*Xylaria venustula*	GU300091	97	OTU7,8			4					4
5219	*Daldinia eschscholzii*	AB284189	99	OTU18			1					1
5160,5063,5283	Xylariaceae	AB440120	99	OTU4	7			3				10
5220,5256,5327	*Xylaria badia*	GU322446	99	OTU3			2		4			6
5336	*Xylaria multiplex*	GU300099	86						1			1
5078,5229	*Nodulisporium* sp.	GQ334429	99	OTU17			1					1
5341,5241,5250	*Annulohypoxylon* sp.	FM209456	98	OTU16			2		1		1	4
Individual number					50	41	26	38	8	13	41	217
evenness index (E′)					0.94	0.83	0.75	0.82	0.85	0.86	0.66	
Shannon Index(H′)					1.68	1.33	1.46	1.47	1.37	1.38	0.92	

**Table 4:** The similarity index of xylariaceous fungal in seven *Dendrobium* species.

Plant species	*D. nobile*	*D. fimbriatum*	*D. chrysotoxum*	*D. chrysanthum*	*D. falconeri*	*D. aphyllum*	*D. crystallinum*
*D. nobile*		0.18	0	0.50	0.36	0.18	0.10
*D. fimbriatum*			0.33	0.55	0	0.20	0.67
*D. chrysotoxum*				0.15	0.33	0.33	0.36
*D.chrysanthum*					0.18	0.55	0.60
*D. falconeri*						0.20	0.22
*D. aphyllum*							0.22

### Phylogenetic Analyses

From each morphotype group, we selected 1–3 representative xylariaceous strains to analyze their ITS, nrLSU and β-tubulin sequences. We newly acquired 53 ITS, 49 nrLSU, and 36 β-tubulin sequences; the GenBank accession numbers are shown in Table S1. Based on the primary ITS and β-tubulin blast results from the GenBank database, of the 217 newly isolated xylariaceous endophytes, 63 strains were closely matched to sequences from *Nemania* spp., 6 strains were similar to sequences from *Annulohypoxylon* spp., 4 strains were closely related to sequences from *Nodulisporium* spp., and the remaining 144 strains were closely matched to the sequences from members of the *Xylaria* genus (Table 3).

Phylogenetic analyses of the newly isolated xylariaceous strains were performed based on the ITS, nrLSU, and β-tubulin sequences. For the ITS analysis, only 5.8S-ITS2 was used because of the high divergence of the ITS1 region.

The 5.8S-ITS2 dataset consisted of 125 aligned sequences, containing 339 characters, and 89 were phylogenetically informative sites. The majority rule tree from the Bayesian analyses is shown in Figure 2. The newly isolated xylariaceous endophytes were clustered into several taxonomic groups of the family, representing at least 18 OTUs (Figure 2). Three OTUs (OTU16-OTU18) belong to members of the *Xylaria* sister genera in Xylariaceae, including *Annulohypoxylon, Nodulisporium*, and *Daldinia*. The strains identified as *Nemania* were separated into two OTUs (OTU4 and OTU15) in the tree, and the other 13 OTUs mainly represent species of the *Xylaria* genus. The phylogenetic analysis and blast search showed that approximately 19 species of Xylariaceae were isolated and that *Nemania diffusa*, *N. bipapillata, X. arbuscula,* and *X. grammica* were the most commonly isolated xylariaceous endophytes from the roots of *Dendrobium* from southwestern China.

**Figure 3:**
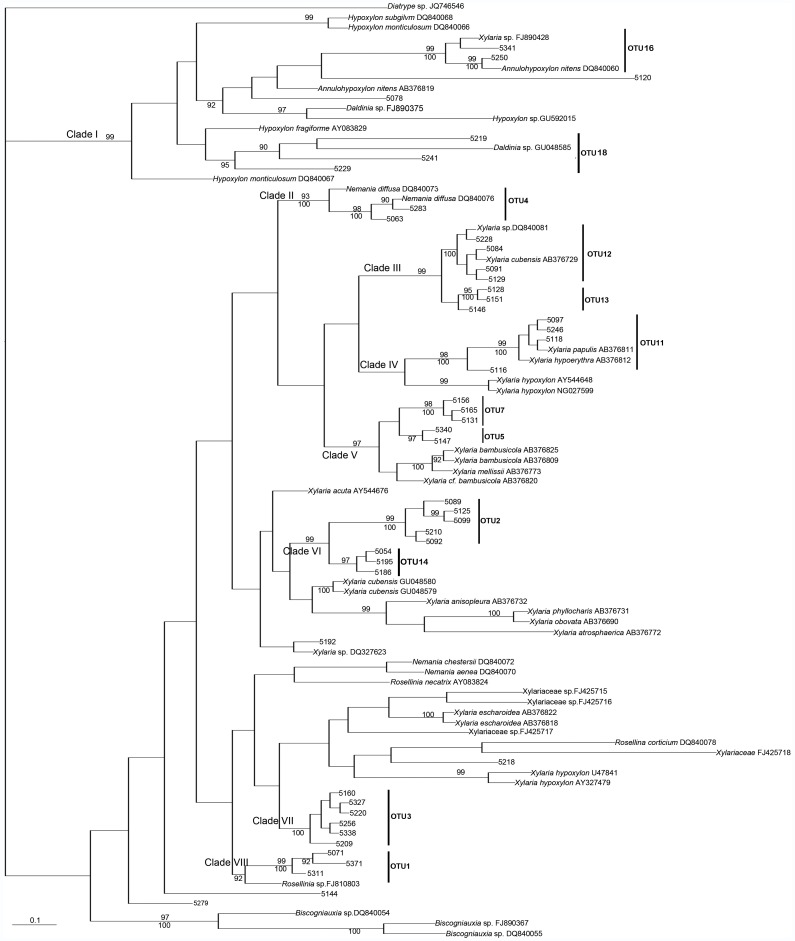
Bayesian 90% majority-rule tree for endophytic Xylariaceae isolated from *Dendrobium* from nrLSU rDNA sequences. Numbers above branches indicate posterior probabilities (≥90%) and numbers below branches are bootstrap values (≥50%) from 1000 replicates.

The alignment of the nrLSU sequence data, consisting of 93 sequences, contained 780 nucleotides and 133 parsimony-informative sites. The phylogenetic trees generated using Bayesian analyses (BA) and maximum parsimony (MP) analyses have similar topology, thus only the tree from the BA is shown in Figure 3. The tree constructed from the LSU sequences clustered all the newly isolated Xylariaceae fungi into 8 clades, representing 12 OTUs, which correspond to OTUs 1–5, 7, 11–14, 16, and 18 in the ITS tree. OTUs 6, 8, 9, 10, 15, and 17 in the ITS tree do not form a well-supported single group in the nrLSU phylogenetic tree. In addition, the members of each OTU in the nrLSU tree are similar to those in the 5.8S-ITS2 tree, but their placement is not exactly consistent with that in the 5.8S-ITS2 tree.

**Figure 4:**
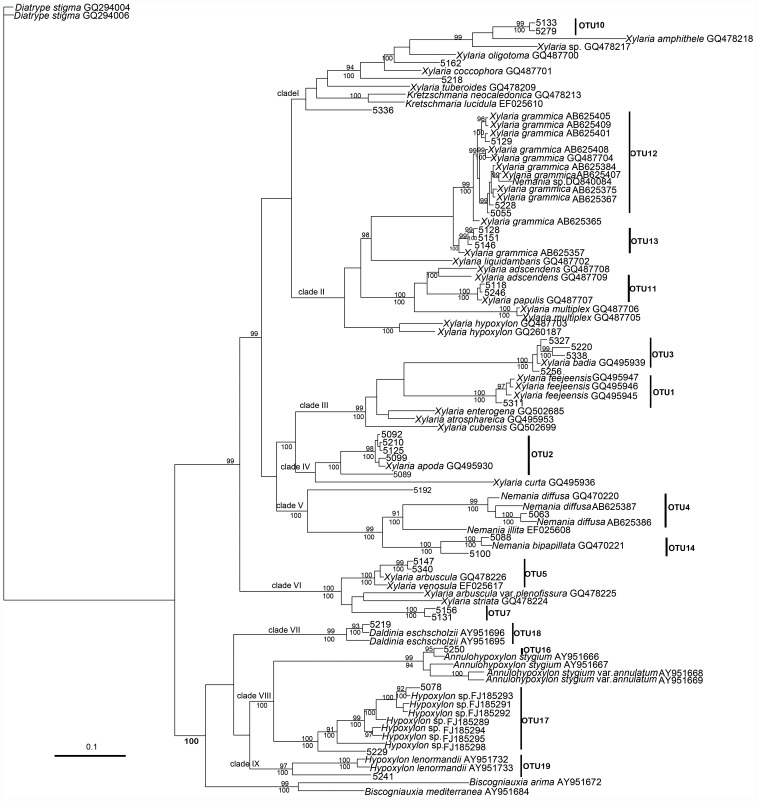
Bayesian 90% majority-rule tree for endophytic Xylariaceae isolated from *Dendrobium* from beta-tubulin sequences. Numbers above branches indicate posterior probabilities (≥90%) and numbers below branches are bootstrap values (≥50%) from 1000 replicates.

The β-tubulin matrix contained 100 aligned sequences, 946 total characters, and 355 parsimony-informative characters. The resulting phylogeny (Figure 4) reveal that all the xylariaceous taxa analyzed formed two large groups, representing members of the *Xylaria* genus (with *Nemania* nested in it) and other related genera in Xylariaceae (e.g., *Hypoxylon, Annulohypoxylon*, and *Daldinia*). The xylariaceous endophytes formed 9 clades and represented 15 OTUs. We were unable to amplify the β-tubulin region for the taxa of OTU6 and OUT15 in the ITS tree.

Based on the phylogenetic analyses, each *Dendrobium* species was associated with at least 3 OTUs of xylariaceous endophytes (Table 3 and Figure 2). The endophytic xylariaceous fungi associated with *D. nobile* are members of 6 OTUs (OTUs 4, 5, 7, 10, 13, and 15), and those associated with *D. chrysotoxum* are members of 9 OTUs (OTUs 3, 6, 8, 9, 11, 12, and 16–18). OTU15 (*Nemania* spp.) was found in four of seven *Dendrobium* species (*D. nobile, D. fimbriatum*, *D. chrysanthum* and *D. crystallinum*), and *X. arbuscula* (OTU5) and *X. grammica* (OTU12) were also commonly detected in four to five species of *Dendrobium*. In addition, xylariaceous OTU7 and OTU13 were restricted to associations with *D. nobile*, and OTU2 and OTU8–9 were each also restricted to a single *Dendrobium* species, *D. chrysotoxum* and *D. fimbriatum*, respectively.

Although several *Dendrobium* species were found to share a common *Xylaria* species in this study, the dominant *Xylaria* taxa in each *Dendrobium* species were different except for *D. crystallinum* and *D. chrysanthum*. The dominant *Xylaria* of *D. nobile*, *D. fimbriatum*, *D. chrysotoxum, D. falconeri*, and *D. aphyllum* were OTU7 (28%), OTU2 (48.78%), OTU11 (53.85%), OTU3 (50%) and OTU14 (46.15%), respectively. *D. crystallinum* and *D. chrysanthum* presented the same dominant xylariaceous fungi (OTU 15, *Nemania* spp.), and the rate was 58.53% and 42.12%, respectively.

## Discussion

Endophytic Xylariaceae have been reported as common, sometimes dominant fungi within the microbiological flora of the roots of tropical Orchidaceae plants [34], [35], [39]. Our analyses showed that xylariaceous fungi, as non-mycorrhizal fungi, are very abundant and diverse in the roots of *Dendrobium* species.

Morphology is the most reliable way for identification of xylariaceous fungi [27]. However, it is difficult to delimitate endophytes (including endophytic xylariaceous) based on morphology because most of endophytes do not produce sexual states in culture [16]. Rogers [19] and Callan & Rogers [20] have provided a useful method for anamorph production and a key to the identification of certain European xylariaceous species in culture. We attempted to induce the formation of conidia in culture on 2.5% oatmeal agar media (OA) under irregular light, but only a few strains formed stroma after 6 weeks. The possible explains could be that the strains we isolated do not form conidiophores or conidia under the currently cultural condition or that the production of their conidia requires more than 6 weeks of incubation time. Another reason is that, although our strains are similar to the species originally described from Europe, possibly, they are endemic to Asia and the asexual characteristics of the European species are dissimilar. Petrini & Petrini (1985) have recognized that colony growth rate, color, and stromatic structure formation as relatively stable diagnostic characters for delimiting xylariaceous species at the intragenus level in culture compared to conidiophore morphology and the size and shape of conidia [16]. The validity of morphotypes as taxonomic groups was further verified by ribosomal DNA sequences (ITS) [40]. In the present study, some xylariaceous strains isolated in our study were preliminarily grouped into morphotypes by the colony characters and hyphal strands.

Molecular techniques have become the most powerful and indispensable tools in identification, community diversity, and phylogeny studies of endophytic fungi, including endophytic *Xylaria* species [22]–[26]. Meanwhile, KoKo et al. (2011) also showed that identifying endophytes by blasting with GenBank sequences often resulted in the wrong naming of taxa and therefore any results must be treated with caution [41]. Thus, the reference sequences downloaded from GenBank database for blasting and conducting phylogenetic tree in this study were mostly published in previous studies [25]–[27], [42]. Among DNA markers, the ITS region is most commonly used for species delimitation. Recently, the ITS region has been confirmed to be applicable as a fungal barcode, and it is able to identify successfully a broad range of fungi (approximately 70%) [43]. However, Hsieh et al. reported that ITS sequences were unsuitable for addressing phylogenetic relationships in Xylariaceae at the genus level [25]. Consistent with the previous study [25], we found that it was difficult to align the ITS sequences of the taxa due to the high variability of the ITS1 region. Thus, only the 5.8S-ITS2 region was selected for phylogenetic tree construction. The intra-group topology of the tree generated using 5.8S- ITS2 was similar to those based on LSU and β-tubulin except for a low intergroup resolution, because there are relatively fewer characters contained in the 5.8S-ITS2 dataset compared to other two datasets.

The mycorrhizal specificity of orchids has been reported, and mycorrhizal symbiosis partners were usually found to be fungi belonging to Tulasnellaceae, Ceratobasidiaceae, Sebacinales, and ectomycorrhizal Russulaceae and Tuberaceae [44], [45]. However, the specificity of non-mycorrhizal endophytes, particularly xylariaceous fungi, in orchid plants is not fully understood. It is difficult to determine the level of specificity in the interaction between host plants and their endophytic fungi because of the endophytic diversity and the limitation of the current research methods [39], though a number of previous studies have demonstrated that endophytic fungi host-specificity was common [46], [47]. In the present study, we found that some endophytic Xylariaceae were exclusively detected in specific host plants. For example, OTU7 and OTU13 were uniquely detected in the roots of *D. nobile*, whereas OTU2 and OTU8-9 were specifically found in the roots of *D. chrysotoxum* and *D. fimbriatum*, respectively. Based our limited samples, these findings might imply that non-mycorrhizal endophytic Xylariaceae have host specificity and selectivity to some extent. However, the endophyte community is dynamic, and seasonal changes and host age will likely influence the species composition [48]. Therefore, the endophytic xylariaceous specificity of Orchidaceae should be estimated using both cultural-dependent and culture-independent methods, such as environmental PCR, for a given habitat and growth stage and sample size.

Moreover, the present study indicated that the dominant Xylariaceae taxa in each *Dendrobium* species were different, though several *Dendrobium* species shared a common *Xylaria* species. *D. fimbriatum* and *D. chrysotoxum* have a close phylogenetic relationship [49], while in the present study, the two species were colonized by different xylariaceous fungi, indicating that different host plants in the same habitat might have preference and selectivity for their fungal partner.

Xylariaceous fungi are highly abundant in the medicinal plants of *Dendrobium*, but their ecological function has remained unclear to date. Davis et al. [33] have mentioned that xylariaceous fungi in liverworts might be mutualistic with their host, although the association of *Xylaria* as mycorrhizal fungi in orchid plants has yet to be confirmed. Moreover, endophytic fungi play an important role in regulating the production of secondary metabolites in their host plants and may increase the concentrations of total alkaloids and polysaccharides in *Dendrobium* [50]. Accordingly, the secondary metabolites of these rich xylariaceous taxa in *Dendrobium* should be widely explored, and the relationship between the endophytic *Xylaria* species and the quality of *Dendrobium* as a medicinal herb need to be extensively investigated.

## Materials and Methods

### Dendrobium Plants

Healthy seven *Dendrobium* species, *D. nobile, D. fimbriatum, D. chrysotoxum, D. chrysanthum, D. falconeri, D. aphyllum* and *D. crystallinum*, were collected from an area of tropical rainforest at the Xishuangbanna, Jinghong City, Yunnan Province, southwestern China in 2009. Plants were identified based on the description of Chen et al [51]. The wild plant samples we studied are very precious medicinal plants and nearly extinct in China. Based on the consideration on the conservation of both the plant and fungal resources, we had to collect only a few individuals per species.

### Fungal Isolation and Identification

Endophytic fungi were obtained after surface-sterilization of the plant tissues [38]. Symptomless roots of each plant species were rinsed in a sequence distilled water for 1 min, 75% ethanol for 30 s, 3% NaClO for 1 min, and 75% ethanol for 30 s and then rinsed in sterile distilled water three times. Each root was then cut into 2–3 mm sections and placed on potato dextrose agar (PDA). The isolates were subcultured on OA medium for morphological identification and conidial production. The plant samples and fungal isolations are listed in Table 1.

The identification of a representative endophyte culture as a xylariaceous fungus was initially based on the characters of colony and mycelium, according to the description of Petrini & Petrini [16] and Callan & Rogers [20]. These xylariaceous cultures were preliminarily classified into different morphotypes. In addition, internal transcribed spacer region (ITS), large subunit ribosomal RNA (nrLSU), and β-tubulin sequences were employed for identification.

### DNA Extraction, PCR Amplification and Phylogenetic Analyses

Genomic DNA was extracted from pure mycelia using the E.Z.N.A. Fungal DNA kit (Omega Bio-Tek, Doraville, GA, USA) according to the manufacturer’s protocol. The PCR amplification followed Chen et al. [37]. The primer pairs ITS 1 and ITS 4 for the ITS sequences, primer pairs LROR and LR7 for the nrLSU sequences [52] and primer pairs T1 and T22 for β-tubulin [53] were used. The sequences obtained were blasted against the GenBank sequences of known members of Xylariaceae, and selected related sequences with high similarity were downloaded for further phylogenetic analyses. The sources for the reference sequences used in the phylogenetic analyses are mostly those reported by Hsieh et al. [25], [42], Okane et al. [23], [26], and Tang et al. [24]. All the sequences were aligned using Clustal X 1.83 [54]. The phylogenetic analyses were performed using MEGA5 [55] for maximum parsimony analyses (MP) and MrBayes 3.1.2. [56] for Bayesian analyses (BA) on same dataset of 5.8S-ITS2, LSU, and β-tubulin. For the BA analysis, the best-fitted evolutionary model was estimated using MrModeltest version 2.3 [57]. The Bayesian analyses was performed with uniform priors for 2,000,000–5,000,000 generations and sampled every 1000 generations. A probability of 90% was considered significant.

### Data Statistical Analyses

The isolation ration (F) was calculated by the formula F = number of fragments colonized by fungi/total number of fragments examined. To compared the specificity and distribution of the xylariaceous species in the seven *Dendrobium* species, the Shannon-Weiner diversity index (H′) and evenness index (E′) were calculated based on the number of OTUs using the following two formulas, respectively: H′ = −Σ(pi ×lnpi) and E′ = H′/lnS (S indicates the total number of species) [58].

## Supporting Information

Table S1 **Genbank accession numbers of sequences obtained in our lab (5054–5371) and other sequences used in phylogenetic analysis.**
(DOC)
