# Supplementary material for: Diversity and Taxonomy of Endophytic Xylariaceous Fungi from Medicinal Plants of Dendrobium (Orchidaceae)
Source: PLoS One. 2013 Mar 5;8(3):e58268. doi: 10.1371/journal.pone.0058268 (PMC3589337; doi:10.1371/journal.pone.0058268)
Supplement: Table S1 — Genbank accession numbers of sequences obtained in our lab (5054–5371) and other sequences used in phylogenetic analysis. (DOC) [file pone.0058268.s001.doc]

**Table S1.** Genbank accession numbers of sequences obtained in our lab (5054-5371) and other sequences used in phylogenetic analysis

| **Fungal taxa** | **Culture** | **ITS** | **nrLSU** | **Beta-tubulin** |
| --- | --- | --- | --- | --- |
| *Xylaria* sp. | 5054 | JQ862654 | JQ862604 |  |
| *Xylaria* sp. | 5055 | JQ862682 |  | JX868520 |
| *Nemania bipapillata* | 5063 | JQ862655 | JQ862605 | JX868523 |
| *Xylaria feejeensis* | 5071 | JQ862701 | JQ862606 |  |
| *Nodulisporium* sp. | 5078 | JQ862656 | JQ862607 | JX868524 |
| *Xylaria grammica* | 5084 | JQ862657 | JQ862608 |  |
| *Nemania bipapillata* | 5088 | JQ862658 |  | JX868525 |
| *Xylaria acuta* | 5089 |  | JQ862609 | JX868551 |
| *Xylaria grammica* | 5091 | JQ862659 | JQ862610 |  |
| *Xylaria acuta* | 5092 |  | JQ862611 | JX868526 |
| *Xylaria* sp. | 5097 | JQ862699 | JQ862612 |  |
| *Xylaria apoda* | 5099 | JQ862660 | JQ862613 | JX868527 |
| *Nemania bipapillata* | 5100 | JQ862661 | JQ862614 | JX868528 |
| *Xylaria* cf. *papulis* | 5116 |  | JQ862615 |  |
| *Xylaria* cf. *papulis* | 5118 | JX868517 | JQ862616 | JX868529 |
| *Annulohypoxylon* sp. | 5120 | JQ862703 | JQ862617 |  |
| *Xylaria apoda* | 5125 | JQ862662 | JQ862618 | JX868530 |
| *Xylaria grammica* | 5129 | JQ862683 | JQ862620 | JX868532 |
| *Xylaria* sp. | 5128 | JQ862680 | JQ862619 | JX868531 |
| *Xylaria* sp. | 5131 | JQ862681 | JQ862621 | JX868547 |
| *Xylaria* sp. | 5133 | JQ862698 | JQ862622 | JX868521 |
| *Xylaria* sp. | 5144 | JQ862663 | JQ862623 |  |
| *Xylaria* sp. | 5146 | JQ862697 | JQ862624 | JX868533 |
| *Xylaria* sp. | 5147 | JQ862664 | JQ862625 | JX868534 |
| *Xylaria grammica* | 5151 | JQ862665 | JQ862626 | JX868535 |
| *Xylaria* sp. | 5156 | JX868518 | JQ862627 | JX868552 |
| *Xylaria* sp. | 5160 | JQ862666 | JQ862628 |  |
| *Xylaria* sp. | 5162 | JQ862686 |  | JX868536 |
| *Xylaria* sp. | 5163 | JQ862679 |  |  |
| *Xylaria* sp. | 5165 | JQ862667 | JQ862629 |  |
| *Xylaria* sp. | 5186 | JQ862668 | JQ862630 |  |
| *Nemania* sp. | 5192 | JQ862669 | JQ862631 | JX868553 |
| *Xylaria* sp. | 5195 | JQ862670 | JQ862632 |  |
| *Xylaria* sp. | 5209 | JQ862671 | JQ862633 |  |
| *Xylaria apoda* | 5210 | JQ862672 | JQ862634 | JX868548 |
| *Xylaria grammica* | 5213 | JQ862684 |  |  |
| *Xylaria* sp. | 5218 | JQ862673 | JQ862635 | JX868549 |
| *Xylaria* sp. | 5219 | JQ862675 | JQ862636 | JX868522 |
| *Xylaria acuta* | 5220 | JQ862676 | JQ862637 | JX868537 |
| *Xylaria grammica* | 5228 | JQ862677 | JQ862638 | JX868538 |
| *Hypoxylon* sp. | 5229 | JQ862678 | JQ862639 | JX868539 |
| Hypoxylon sp. | 5241 | JQ862640 | JQ862706 | JX868540 |
| *Xylaria* cf. *papulis* | 5246 | JQ862700 | JQ862641 | JX868541 |
| Xylariaceae | 5250 | JQ862704 | JQ862642 | JX868542 |
| *Xylaria* cf. *badia* | 5256 | JQ862687 | JQ862643 | JX868543 |
| *Xylaria bambusicola* | 5268 | JQ862694 | JQ862644 |  |
| *Xylaria* sp. | 5279 |  | JQ862645 | JX868544 |
| *Xylaria* sp. | 5283 | JQ862688 | JQ862646 |  |
| Xylariaceae | 5306 | JQ862674 |  |  |
| *Xylaria* sp. | 5311 | JQ862689 | JQ862647 | JX868545 |
| *Xylaria* sp. | 5327 | JQ862690 | JQ862648 | JX868519 |
| *Nemania* cf. *bipapillata* | 5336 | JQ862691 | JQ862649 | JX868546 |
| *Xylaria* sp. | 5338 | JQ862692 | JQ862650 | JX868550 |
| *Xylaria* sp. | 5340 | JQ862693 | JQ862651 | JX868554 |
| Xylariaceae | 5341 | JQ862702 | JQ862652 |  |
| *Xylaria badia* | 5351 | JQ862696 |  |  |
| Xylaria feejeensis | 5371 | JQ862695 | JQ862653 |  |
| Xylariaceae |  |  | FJ425715 |  |
| Xylariaceae |  |  | FJ425716 |  |
| Xylariaceae |  |  | FJ425717 |  |
| Xylariaceae |  |  | FJ425718 |  |
| *Annulohypoxylon nitens* |  |  | DQ840060 |  |
| *Annulohypoxylon nitens* |  |  | AB376819 |  |
| *Annulohypoxylon cohaerens* |  | EF026140 |  |  |
| *Annulohypoxylon multiforme* |  | GU062284 |  |  |
| *Annulohypoxylon stygium* |  |  |  | AY951666 |
| *Annulohypoxylon stygium* |  |  |  | AY951667 |
| *Annulohypoxylon stygium* |  |  |  | AY951668 |
| *Annulohypoxylon stygium* |  |  |  | AY951669 |
| *Biscogniauxia latirima* |  | EF026135 |  |  |
| *Biscogniauxia* sp. |  |  | FJ890367 |  |
| *Biscogniauxia* sp. |  |  | DQ840054 |  |
| *Biscogniauxia* sp. |  |  | DQ840055 |  |
| *Biscogniauxia philippinensis var. microspora* |  | EF026136 |  |  |
| *Biscogniauxia arima* |  |  |  | AY951672 |
| *Biscogniauxia mediterranea* |  |  |  | AY951684 |
| *Daldinia eschscholzii* |  |  |  | AY951696 |
| *Daldinia eschscholzii* |  | AB284189 |  | AY951695 |
| *Daldinia* sp. |  | GU222391 | FJ890375 |  |
| *Daldinia* sp. |  |  | GU048585 |  |
| *Diatrype disciformis* |  | AJ390410 |  |  |
| *Diatrype* sp. |  |  | JQ746546 |  |
| *Diatrype stigma* |  |  |  | GQ294004 |
| *Diatrype stigma* |  |  |  | GQ294006 |
| *Hypoxylon fragiforme* |  | AY618235 |  |  |
| *Hypoxylon fragiforme* |  | JN979420 |  |  |
| *Hypoxylon fragiforme* |  | FN435637 |  |  |
| *Hypoxylon monticulosum* | AF-04 |  | DQ840066 |  |
| *Hypoxylon monticulosum* | M050 |  | DQ840067 |  |
| *Hypoxylon fragiforme* |  |  | AY083829 |  |
| *Hypoxylon* sp. |  |  | GU592015 | FJ185293 |
| *Hypoxylon* sp. |  |  |  | FJ185291 |
| *Hypoxylon* sp. |  |  |  | FJ185292 |
| *Hypoxylon* sp. |  |  |  | FJ185294 |
| *Hypoxylon* sp. |  |  |  | FJ185295 |
| *Hypoxylon* sp. |  |  |  | FJ185289 |
| *Hypoxylon* sp. |  |  |  | FJ185298 |
| *Hypoxylon lenormandii* |  |  |  | AY951732 |
| *Hypoxylon lenormandii* |  |  |  | AY951733 |
| *Hypoxylon subgilvum* |  |  | DQ840068 |  |
| *Kretzschmaria neocaledonica* |  |  |  | GQ478213 |
| *Kretzschmaria lucidula* |  |  |  | EF025610 |
| *Nemania aenea* |  |  | DQ840070 |  |
| *Nemania bipapillata* |  | EU678662 |  |  |
| *Nemania bipapillata* |  | AY541610 |  | GQ470221 |
| *Nemania chestersii* |  |  | DQ840072 |  |
| *Nemania diffusa* |  | FJ438909 | DQ840073 | GQ470220 |
| *Nemania diffusa* |  |  |  | AB625387 |
| *Nemania diffusa* |  | DQ658238 | DQ840076 | AB625386 |
| *Nemania illita* |  |  |  | EF025608 |
| *Nemania* sp. |  |  |  | DQ840084 |
| *Nodulisporium* sp. |  | AF201751 |  |  |
| *Nodulisporium* sp. |  | GQ334429 |  |  |
| *Rosellinia* sp. |  |  | FJ810803 |  |
| *Rosellinia corticium* |  |  | DQ840078 |  |
| *Rosellinia necatrix* |  |  | AY083824 |  |
| *Xylaria acuta* |  |  | AY544676 |  |
| *Xylaria adscendens* |  | GU300101 |  | GQ487708 |
| *Xylaria adscendens* |  | GU322432 |  | GQ487709 |
| *Xylaria allantoidea* |  | AY909005 |  |  |
| *Xylaria allantoidea* |  | GU324743 |  |  |
| *Xylaria amphithele* |  |  |  | GQ478218 |
| *Xylaria anisopleura* |  |  | AB376732 |  |
| *Xylaria apodia* |  | GU322437 |  | GQ495930 |
| *Xylaria arbuscula* |  | JN601145 |  | GQ478226 |
| *Xylaria arbuscula* |  |  |  | GQ478225 |
| *Xylaria atrosphaerica* |  | GU322459 | AB376772 | GQ495953 |
| *Xylaria badia* |  | GU322446 |  |  |
| *Xylaria badia* |  | DQ322138 |  |  |
| *Xylaria badia* |  | DQ322137 |  |  |
| *Xylaria bambusicola* |  | GU300088 | AB376809 |  |
| *Xylaria bambusicola* |  | EF026123 | AB376825 |  |
| *Xylaria* cf*.arbuscula* |  | FN689806 |  |  |
| *Xylaria* cf*.arbuscula* |  | FN689807 |  |  |
| *Xylaria* cf*.bambusicola* |  |  | AB376820 |  |
| *Xylaria badia* |  |  |  | GQ495939 |
| *Xylaria berteri* |  | GU324750 |  |  |
| *Xylaria carpophila* |  | F908806 |  |  |
| *Xylaria castorea* |  | JF908802 |  |  |
| *Xylaria castorea* |  | GU324751 |  |  |
| *Xylaria coccophora* |  | GU300093 |  | GQ487701 |
| *Xylaria crozonensis* |  | GU324748 |  |  |
| *Xylaria curta* |  | GU322444 |  | GQ495936 |
| *Xylaria cubensis* |  |  | GU048580 | GQ502699 |
| *Xylaria cubensis* |  |  | AB376729 |  |
| *Xylaria cubensis* |  |  | GU048579 |  |
| *Xylaria digitata* |  | AY909006 |  |  |
| *Xylaria digitata* |  | GU322456 |  |  |
| *Xylaria enterogena* |  |  |  | GQ502685 |
| *Xylaria escharoidea* |  |  | AB376822 |  |
| *Xylaria escharoidea* |  |  | AB376818 |  |
| *Xylaria feejeensis* |  | HM992808 |  | GQ495945 |
| *Xylaria feejeensis* |  | GU322454 |  | GQ495947 |
| *Xylaria feejeensis* |  | GU322452 |  | GQ495946 |
| *Xylaria frustulosa* |  | GU322450 |  |  |
| *Xylaria globosa* |  | AY909008 |  |  |
| *Xylaria grammica* |  | GU300097 |  | AB625384 |
| *Xylaria grammica* |  | AB524025 |  | AB625367 |
| *Xylaria grammica* |  | DQ322145 |  | AB625375 |
| *Xylaria grammica* |  |  |  | AB625405 |
| *Xylaria grammica* |  |  |  | AB625357 |
| *Xylaria grammica* |  |  |  | AB625365 |
| *Xylaria grammica* |  |  |  | AB625401 |
| *Xylaria grammica* |  |  |  | AB625408 |
| *Xylaria grammica* |  |  |  | AB625409 |
| *Xylaria grammica* |  |  |  | AB625407 |
| *Xylaria grammica* |  |  |  | GQ487704 |
| *Xylaria hypoxylon* |  | AM993138 | AY327479 | GQ260187 |
| *Xylaria hypoxylon* |  | AY909010 | NG027599 | GQ487703 |
| *Xylaria hypoxylon* |  | GU300095 | AY544648 |  |
| *Xylaria hypoxylon* |  | GU300096 | U47841 |  |
| *Xylaria hypoerythra* |  |  | AB376812 |  |
| *Xylaria ianthinovelutina* |  | GU322441 |  |  |
| *Xylaria intracolorata* |  | GU324741 |  |  |
| *Xylaria juruensis* |  | GU322439 |  |  |
| *Xylaria laevis* |  | GU324747 |  |  |
| *Xylaria liquidambaris* |  | GU300094 |  | GQ487702 |
| *Xylaria longiana* |  | JF908807 |  |  |
| *Xylaria luteostromata* var. *macrospora* |  | GU324739 |  |  |
| *Xylaria mali* |  | GU355650 |  |  |
| *Xylaria mellissii* |  |  | AB376773 |  |
| *Xylaria multiplex* |  | GU300098 |  | GQ487706 |
| *Xylaria multiplex* |  | GU300099 |  | GQ487705 |
| *Xylaria obovata* |  |  | AB376690 |  |
| *Xylaria oligotoma* |  | GU300092 |  | GQ487700 |
| *Xylaria papulis* |  | GU300101 | AB376811 |  |
| *Xylaria papulis* |  |  |  | GQ487707 |
| *Xylaria persicaria* |  | AY909022 |  |  |
| *Xylaria phyllocharis* |  |  | AB376731 |  |
| *Xylaria plebeja* |  | GU324740 |  |  |
| *Xylaria polymorpha* |  | FN689809 |  |  |
| *Xylaria polymorpha* |  | GU322460 |  |  |
| *Xylaria regalis* |  | GU324745 |  |  |
| *Xylaria scruposa* |  | GU322458 |  |  |
| *Xylaria striata* |  |  |  | GQ478224 |
| *Xylaria* sp. |  |  | FJ890428 |  |
| *Xylaria* sp. |  |  | DQ840081 | GQ478217 |
| *Xylaria* sp. |  |  | DQ327623 |  |
| *Xylaria tuberoides* |  |  |  | GQ478209 |
| *Xylaria venustula* |  | GU300091 |  |  |
| *Xylaria venosula* |  |  |  | EF025617 |
| *Xylariaceae* sp. |  |  | FJ425715 |  |
| *Xylariaceae* sp. |  |  | FJ425716 |  |
| *Xylariaceae* sp. |  |  | FJ425717 |  |
